# Editorial: The Importance of Diversity in Precision Medicine Research

**DOI:** 10.3389/fgene.2020.00875

**Published:** 2020-08-26

**Authors:** Jessica N. Cooke Bailey, William S. Bush, Dana C. Crawford

**Affiliations:** ^1^Department of Population and Quantitative Health Sciences, Cleveland Institute for Computational Biology, Case Western Reserve University, Cleveland, OH, United States; ^2^Department of Genetics and Genome Sciences, Case Western Reserve University, Cleveland, OH, United States

**Keywords:** precision medicine, diversity, genomics, personalized medicine, research participant, genetic ancestry, polygenic risk scores, social determinants of health

Personalized or precision medicine is meant to distinguish tailored treatment from trial and error. The contemporary concept has evolved to specifically include the ‘omic profile of a patient in the prevention, diagnosis, and treatment of disease. Rapid genomic discoveries made possible through genome-wide association studies (GWAS) coupled with decreasing costs of sequencing and genotyping have shifted precision medicine from an academic exercise to clinical reality for some conditions (e.g., Wigle et al., 2017; Claassens et al., 2019; Hamdan et al., 2019; Lim, 2019; Roden, 2019), while others are not far behind. The emergence of electronic health records (EHRs) now makes it possible to both perform population-scale research and effectively deliver personalized medicine to the individual patient through clinical decision support.

While the promise of precision medicine is great, several identifiable gaps exist in current research that limit its reach to all potential patients. One key deficiency is the lack of diversity among biomedical research participants, which limits both the generalizability and availability of genomic-based treatments or prevention strategies. The vast under-representation of diverse populations in genetic/genomic studies (e.g., Sirugo et al., 2019) is highly problematic as genetic information gleaned from one population is not automatically transferrable across populations (Popejoy and Fullerton, 2016). Without sample diversity, signals revealing powerful insights into genetic association and/or drug response can go undetected due to differences in linkage disequilibrium, allele frequencies, and genetic architecture. New initiatives and studies are now in place to ensure the inclusion of traditionally underrepresented groups, defined by race/ethnicity, socioeconomic status and/or position, geography, and age, in genomic research (Bentley et al., 2020). We therefore anticipate a swell of new data and methodologies accelerating the already rapid pace of precision medicine research.

Our goal for this Research Topic was to present original research, commentaries, perspectives, and reviews on the impact and importance of diversity in precision medicine research. Below, we briefly overview the nine accepted manuscripts and the context in which they address this goal.

## Importance of Recruitment and Retention of Diverse Participants

A 2009 analysis of GWAS participants revealed only 4% of DNA samples were from non-European participants (Need and Goldstein, 2009). By 2016, 20% of DNA samples were from non-European samples; however, this more than 2000%-fold increase was mainly due to expansion of studies in primarily East Asian ancestry populations (Popejoy and Fullerton, 2016). Taken together, less than 4% of samples analyzed were from individuals of African and Latin American ancestry, Hispanic people, and native or indigenous peoples, despite that these are the most vulnerable and traditionally underserved populations worldwide (Popejoy and Fullerton, 2016). Inclusion of diverse groups is key to diversifying the pool from which precision medicine can be developed. Discerning factors that influence participation and incorporating these findings into inclusive ascertainment strategies are crucial; efforts must be made to understand ways in which diverse groups can be accessed and invited to participate, as well as to identify motivators and/or barriers affecting willingness to participate and to remain in studies (Perreira et al., 2020). Four publications in this Research Topic addressed the topic “development of culturally-appropriate consent and recruitment strategies for precision medicine research” and “barriers to participation in research (such as access to technology, genomic literacy, concerns for digital data privacy, and factors that impact time or means to participate in research).”

To identify addressable issues and adjust enrollment protocols to improve participation among Hispanics, Nuytemans et al. sought to identify motivators of patients and caregivers affected by Parkinson's disease (PD) to participate in genomic research via surveys administered to patients in the University of Miami Health System's Movement Disorders Clinic, wherein approximately 35% of patients identify as Hispanic. Of the more than 150 self-identified white PD patients and caregivers, approximately 60% of whom were Hispanic, Hispanics and non-Hispanics were equally motivated to participate in genetic research for PD, but Hispanic patients were less likely to be influenced by the promise of scientific advancements. This lack of scientific interest was found to be likely confounded by lower levels of obtained education. The authors suggest that a potential reason for the underrepresentation of Hispanics in genetic research is due to reduced invitations to studies.

Also focused on motivators for research participation within a patient population impacted by a chronic disease, Cuccaro et al. surveyed individuals with multiple sclerosis (MS) participating in a genetic study of MS. The majority of approached study participants (95/101) were willing to participate in the survey; of these, over 80% were Hispanic and female. Survey respondents were asked to identify the primary reasons or motivations for participation. The most frequently cited reason was finding a cure, equally endorsed by Hispanic and non-Hispanic participants; having MS and helping future generations were also highly endorsed motivators, with Hispanics more frequently citing having MS and non-Hispanics more frequently citing finding new/better treatments. Overall, ethnicity was the only significant factor associated with willingness to participate.

The dearth of genetic data available for populations other than those of European descent extends to pharmacogenomics (PGx), the study of genomic information relevant to drug response to tailor dosing. Scherr et al. review challenges to recruiting African American participants in genomic studies and extrapolate these findings to PGx. Consistent with prior reports, their review highlighted African American distrust of the healthcare system, medical research, organization, and researchers as barriers to study participation. Authentic, intentional collaborations between researchers and communities are suggested as a means by which to begin overcoming distrust. Another overarching barrier was lack of knowledge or awareness regarding genomic studies. To reduce distrust and increase awareness, they suggest transparent and clearly described study protocols, educational messaging, and recruitment efforts that directly address existing attitudes and beliefs of distrust. Importantly, there was no evidence of lack of interest in research study participation; conversely, they found that African Americans are aware that participation in medical research is crucial to medical and scientific advancement. Thus, Scherr et al. suggest a focused approach to recruiting African American research study participants, including messaging that highlights altruism.

Alzheimer disease (AD) is another common, complex disease with later-in-life onset and for which most genetic and genomic studies to date have focused on individuals of European descent (e.g., Beecham et al., 2017). Feliciano-Astacio et al. describe the ascertainment approach applied in the Puerto Rico Alzheimer Disease Initiative (PRADI), a multisource recruitment effort to increase participation by Puerto Ricans in genomic research of AD, which currently has >670 participants. PRADI's successful recruitment was attained by establishing strong community engagement relationships and tailored recruitment of AD patients and families across multiple sites in Puerto Rico. Focused and deliberate recruitment efforts such as these will help ensure the inclusion of Hispanic and Latino populations in future precision medicine research efforts.

## Population diversity and Gene Expression

One publication in this Research Topic addressed “statistical methods for genomic data from multiple populations.” A popular statistical method, known as PrediXcan (Gamazon et al., 2015), infers gene expression using genetic data. While now widely used on a variety of datasets derived from many different populations, most gene expression datasets are from majority European-descent populations, and thus construction of reference panels used by PrediXcan are based on European-descent data. Mikhaylova and Thornton evaluate the accuracy of PrediXcan in predicting or inferring gene expression in diverse populations. Using a combination of Genetic European Variation in Disease (Geuvadis) RNA sequencing data and 1000 Genomes Project whole genome sequencing data, Mikhaylova and Thornton demonstrate that the performance of PrediXcan varies by population, with lower performance for African-descent populations compared with others available in the 1000 Genomes Project. The data suggest that prediction models developed using European reference panels are not necessarily transferrable to other populations due to differences in allele frequency, linkage disequilibrium, and genetic admixture.

## Social Determinants of Health and Genetic Association Studies

For complex diseases and traits, genetic variation alone does not sufficiently explain the totality of risk or variation. While this observation is widely accepted, few genetic association studies incorporate important measures of lifestyle, environmental exposures, or social determinants of health associated with disease risk and health disparities. Hollister et al. address this challenge by applying their recently validated algorithm that defines socioeconomic status using electronic health records (Hollister et al., 2017) to a large clinical population of African American patients. All patients were clinically screened for hypertension, a complex condition disproportionately prevalent in African Americans (Fryar et al., 2017) that is independently associated with many common genetic variants and environmental exposures such as diet and socioeconomic status (Aburto et al., 2013; Giri et al., 2019; de las Fuentes et al., 2020; Glover et al., 2020; Hollister et al.). In the work presented herein, Hollister et al. tested for and possibly identified a statistical interaction between education, a recognized social determinant of health, and genetic variants contributing to blood pressure, underscoring the need for additional study of the potentially modifying effects of non-genetic factors for diseases with noted population differences.

## Candidate Gene Variation and Chronic Obstructive Pulmonary Disease

Two publications in this Research Topic addressed “Genomic discovery in non-European populations.” Khanna et al. present a meta-analysis of 14 published studies investigating the association between variants rs4588 and rs7041 in the Vitamin-D binding (*GC*) protein locus and chronic obstructive pulmonary disease (COPD). Both *GC* rs4588 and rs7041 are robustly associated with vitamin D levels in GWAS of mostly European-descent populations (Manousaki et al., 2017; O'Brien et al., 2018). The meta-analysis presented by Khanna et al. include both European- and Asian-descent populations. Both single SNP tests of association for COPD and evaluations of linkage disequilibrium and haplotypes using publicly available genomic and *in silico* data are presented for multiple populations to more fully describe the genetic epidemiology of these loci.

Nandy et al. evaluated the association between serum surfactant protein D (SFTPD) concentration and *SFTPD* rs721917 and chronic obstructive pulmonary disease (COPD) and acute exacerbation COPD (AECOPD). Recent large GWAS of mostly European-descent populations have identified *SFTPD* rs721917 as significantly associated with COPD at genome-wide significance (Hobbs et al., 2017; Sakornsakolpat et al., 2019). Nandy and colleagues identified and meta-analyzed results from eight independent published reports, which included six with serum SFTPD concentrations and three with *SFTPD* rs721917 genotype data for Asian populations from China, Lebanon, and Pakistan. As expected, both COPD and AECOPD were associated with serum SFTPD. However, while *SFTPD* rs721917 was significantly associated with both COPD and AECOPD in this meta-analysis, the direction of effect was opposite of that previously reported by recent GWAS of COPD (Hobbs et al., 2017; Sakornsakolpat et al., 2019). While limited in sample size, this small meta-analysis underscores the importance of generalizing GWAS findings in diverse populations.

## Polygenic Risk Scores and Diverse Populations

One publication in this Research Topic addressed “The use of genetic ancestry for genomic discovery (such as admixture mapping).” Genetic and polygenic risk score studies aggregate cumulative effects across genetic loci; effect sizes are typically estimated from GWAS that have traditionally been performed in samples of European descent. Unfortunately, polygenic risk scores do not always replicate in non-European ancestral groups [reviewed in (Sirugo et al., 2019)]. Focusing on Chinese and Japanese samples, Zhou et al. evaluated lumbar disc degeneration (LDD), another complex, age-related phenotype. The focus of this work was to investigate genetic overlap between LDD and four related risk factors. Strong association between a polygenic LDD score, constructed with weights from European-ancestry studies, and related risk factors was detected. However, phenotype variances explained were lower than in prior European studies, thus, reducing power to detect genetic overlaps. This study again emphasizes the importance of genetic studies inclusive of populations other than Europeans.

Taken together, this Research Topic is composed of nine publications that further emphasize the importance of diversity in precision medicine research and offer solutions to better ensure these translational research efforts are realized in the clinic for all to benefit.

## Author Contributions

JC, WB, and DC conceived the idea for and wrote this editorial. All authors contributed to the article and approved the submitted version.

## Conflict of Interest

The authors declare that the research was conducted in the absence of any commercial or financial relationships that could be construed as a potential conflict of interest.
